# Renal artery ablation instead of pulmonary vein ablation in a hypertensive patient with symptomatic, drug-resistant, persistent atrial fibrillation

**DOI:** 10.1007/s00392-012-0529-y

**Published:** 2012-12-14

**Authors:** Dirk Vollmann, Samuel Sossalla, Marco R. Schroeter, Markus Zabel

**Affiliations:** Abteilung Kardiologie und Pneumologie, Georg-August-Universität Göttingen, Universitätsmedizin, Robert-Koch-Straße 40, 37075 Göttingen, Germany

Sirs:

A 58-year-old female with a history of paroxysmal atrial fibrillation (AF) and essential arterial hypertension (HTN) presented to an external institution with palpitations and progressive symptoms and signs of cardiac decompensation. She had been hospitalized twice for episodes of AF and HTN within the last 2 years and was on a daily medication with metoprolol (95 mg), valsartan (320 mg), hydrochlorothiazide (25 mg), minoxidil (10 mg), and aspirin (100 mg). On admission, blood pressure was 165/100 mmHg, and surface ECG confirmed recurrence of AF with an irregular ventricular rate of 130–150 bpm. Rate control was pursued by up-titration of β-blocker dosage and additional administration of digoxin, and therapeutic anticoagulation was initiated. Recompensation was rapidly achieved giving intravenous loop diuretics. Subsequent echocardiography revealed mild left atrial (LA) dilatation, mild mitral regurgitation, and normal systolic but impaired diastolic left ventricular function. While the exact duration of AF was not known, electrical cardioversion was attempted after exclusion of LA thrombi by transesophageal echocardiography. Stable sinus rhythm, however, could not be achieved, even though the patient underwent another electrical cardioversion attempt after administration of amiodarone (8 g in 8 days). Anticoagulation was continued and the patient was referred to our institution for pulmonary vein ablation.

**Fig. 1:**
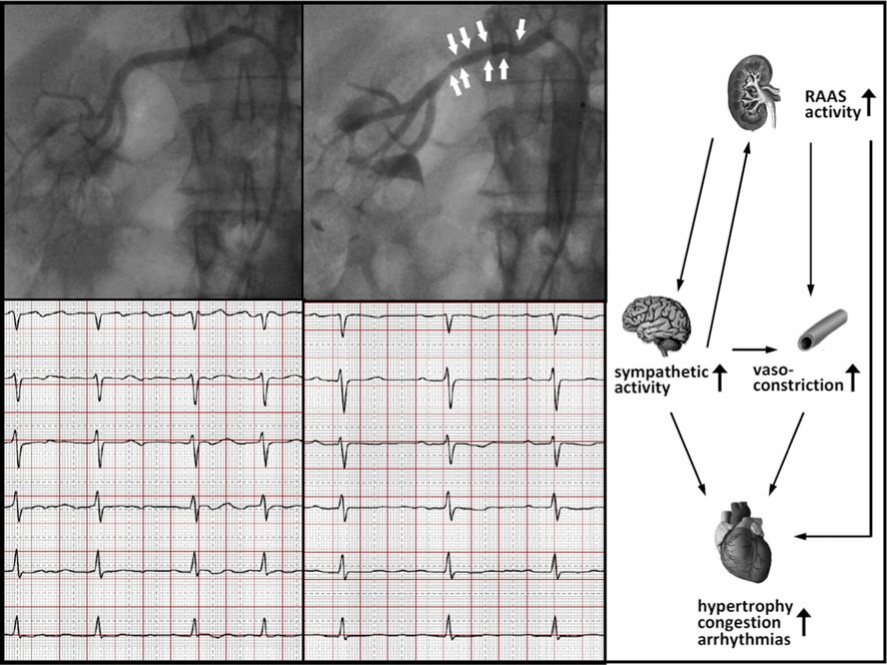
*Top* angiographic imaging of the right renal artery before (*left*) and immediately after (*middle*) application radiofrequency ablation lesions for renal denervation (*arrows* indicate presumed local artery constriction/edema in response to thermal application). *Bottom* surface electrocardiogram (leads V_1_–V_6_) showing coarse atrial fibrillation (*left*) before renal denervation and stable sinus rhythm (*right*) during follow-up. *Right* schematic diagram illustrating the proposed relationship between renal afferent nerve activity, central sympathetic tone, peripheral vascular resistance and cardiac arrhythmias in arterial hypertension (from [13] with modification)

When the patient presented to our center, there were no clinical signs of heart failure but persisting dyspnoea and atypical angina upon mild exertion. Blood pressure was 160/90 mmHg despite a daily medication with metoprolol (190 mg), valsartan (320 mg), minoxidil (10 mg), spironolacton (25 mg) and furosemide (60 mg). Blood pressures at home (self measurements in the sitting position) had varied between 140 and 160 mmHg (systolic) and 90 and 110 mmHg (diastolic). Serum electrolytes and estimated glomerular filtration rate (eGFR) were within normal ranges. The surface ECG showed that the patient was still in AF, now with a resting ventricular rate of 85 bpm. Twenty-four hour Holter monitoring showed persisting AF with a ventricular rate ranging between 79 and 126 bpm. Echocardiography was repeated and confirmed the previous findings, with additional evidence for significant pulmonary hypertension [estimated systolic pulmonary artery pressure (PAP) ~70 mmHg]. Cardiac catheterization revealed post-capillary pulmonary hypertension (PAP systolic 76 mmHg, PAP diastolic 40 mmHg, PAP mean 53; pulmonary capillary wedge pressure 37 mmHg, left ventricular end-diastolic pressure 34 mmHg) and markedly elevated systemic vascular resistance (4,822 dyn × s × m^2^/cm^5^, normal range 1,970–2,390 dyn × s × m^2^/cm^5^). Angiography excluded significant coronary artery disease. Findings were interpreted as diastolic dysfunction on the basis of chronic hypertension and associated hypertensive heart disease. We discussed all findings with the patient, postponed the LA ablation procedure and recommended ablation in the renal arteries for renal denervation as an initial step for intensified treatment of HTN and potential improvement of her AF. Upon written informed consent, a total of 15 radiofrequency ablation lesions (max. 8 W for 120 s, respectively) were applied in the right (eight locations, see Fig. 1) and left (seven locations) renal artery using a Symplicity^®^ Catheter (Medtronic/Ardian Inc., USA). No peri-interventional complications occurred, and the patient was discharged in sinus rhythm after AF had converted spontaneously (under ongoing amiodarone medication).

Three months later, the patient presented to our outpatient clinic and reported marked improvement in symptoms and exercise capacity. She had no more dyspnoea or angina upon exertion and did not report any palpitations. Amiodarone had been stopped 6 weeks earlier because of transient coughing, whereas antihypertensive medication had not been altered. Blood pressure at presentation was 145/80 mmHg. Ninety-six hour Holter monitoring confirmed stable sinus rhythm with a heart rate of 59–102 bpm. Echocardiographic findings were stable except for a significant decrease in estimated systolic PAP (26 mmHg as compared to 70 mmHg 3 months earlier). Digoxin medication was stopped and furosemide was replaced by hydrochlorothiazide (25 mg daily). Another 3 months later the patient was still free of symptoms and in normal sinus rhythm. Ambulatory blood pressure during 24 h Holter monitoring was 111/60 mmHg on average, with a maximum systolic value of 148 mmHg and a maximum diastolic value of 81 mmHg. Blood testing indicated that renal function had remained normal after the ablation procedure (eGFR 84 ml/min), whereas microalbuminuria was diagnosed before renal denervation but excluded during follow-up. Four days of ECG holter monitoring 5 months after amiodarone medication had been stopped confirmed stable sinus rhythm and ruled out asymptomatic AF recurrences. Atrial ectopy had decreased to an average of 2/h as compared to 26/h 3 months earlier. Along with these findings, echocardiography showed a progressive decrease of the left atrial diameter from 45 mm just prior to renal ablation to 40 and 36 mm 3 and 6 months thereafter.

Individual selection of the most appropriate therapy for persistent AF can be cumbersome in subjects that present with heart failure symptoms and cardiovascular co-morbidity. As in the present case, attempts to restore and preserve sinus rhythm appear justified if AF is not long-standing (duration <1 year), if the LA is not significantly enlarged, and if symptoms persist despite sufficient rate control and in the absence of severe structural heart disease.

In our patient, rhythm control was pursued, and in line with current guidelines [1], pulmonary vein ablation was indicated after antiarrhythmic drug treatment (with amiodarone) had failed to maintain sinus rhythm. Despite the given indication, however, it is well appreciated that the success rate of LA ablation is only moderate in persistent AF [1, 2]. In fact, repeated ablation is often required and may in some cases not be restricted to the elimination of triggers (e.g., pulmonary vein isolation) but may also require modification of the perpetuating atrial substrate (e.g., creation of lines of block, targeting sites with complex and fractionated electrograms) [2]. Hypertension and LA dilatation have been identified as strong independent predictors of unsuccessful AF ablation [3]. One study found that LA stiffness, a parameter related to left ventricular diastolic dysfunction, independently predicted unsuccessful AF ablation, whereas LA diameter or LA volume index did not [4].

Catheter ablation in the renal arteries (renal sympathetic denervation) has evolved as a novel therapeutic option for drug-resistant HTN [5, 6]. Its beneficial effects on sympathetic nerve activity [7], the renin-angiotensin-aldosterone system [8], cardiac afterload and left ventricular diastolic dysfunction [9] have also inspired recent interest in its potential impact on AF [10, 11]. Of note, a very recent randomized clinical study performed in subjects with drug-resistant HTN and symptomatic AF demonstrated significantly higher success rates of pulmonary vein ablation after 12 months if the procedure was combined with renal artery denervation [12]. The present case is in line with this study and supports the notion that HTN and its associated changes and sequelae are not only an important substrate but also a hitherto underestimated therapeutic target for persistent AF. Furthermore, it has been speculated that renal artery ablation may have antiarrhythmic effects beyond those that result from a pure reduction of cardiac afterload (Fig. 1). Recent interest has focused on the role of excessive central sympathetic drive in chronic disease and on the effects of its reduction by renal denervation [13]. Specifically, reduction of systemic sympathetic activity has also been proposed as an antiarrhythmic mechanism, by which renal ablation may suppress atrial fibrillation [10, 12] and refractory ventricular tachyarrhythmias [14]. Although the patient in our present case was on high-dose β-blocker medication, one can not exclude that reduction of sympathetic activity also had direct effects on atrial electrophysiology (e.g., reduction of atrial ectopy).

In summary, this case illustrates that in patients presenting with ‘symptomatic, drug-resistant’ AF, careful evaluation and optimized treatment of the underlying substrate (e.g., HTN) should precede consideration for invasive LA ablation procedures. Whether renal denervation in patients with drug-refractory hypertension and atrial fibrillation has an antiarrhythmic effect beyond normalization of blood pressure and cardiac hemodynamics (e.g., due to a reduction of sympathetic activity) remains to be determined.
